# P-1042. Comparison of clinical characteristics and outcomes of patients with candidemia caused by Candida auris versus other Candida species – A retrospective case-control study at a community hospital in the Bronx

**DOI:** 10.1093/ofid/ofae631.1232

**Published:** 2025-01-29

**Authors:** Nada Ahmed, Eris Cani, Lendelle Raymond, Cosmina Zeana

**Affiliations:** Richmond University Medical Center, Staten Island, New York; BronxCare Health System, Bronx, NY; BronxCare Health System, Bronx, NY; BronxCare Health System, Bronx, NY

## Abstract

**Background:**

Conflicting data exists on the mortality rate of *Candida auris* fungemia compared to other *Candida* species. The purpose of this study was to compare the clinical outcomes and characteristics of hospitalized patients with candidemia due to *C. auris* versus non-*C. auris* species.

**Methods:**

This retrospective case-control study included adult patients with microbiologically confirmed candidemia at an urban hospital in New York City from January 2018 to August 2023. Data collected included demographics, medical history, microbiology data, antifungal treatment, in-hospital mortality, length of hospital stay (LoS), recurrence rate, mycological eradication, and discharge status. Descriptive statistics and t test were utilized for continuous variables, and categorical data was analyzed by Fischer’s exact test with significance set at a p-value < 0.05.

**Results:**

Out of 171 candidemia cases, 17% were due to *C. auris* and 83% were due to other *Candida* species, mainly *C. albicans* (29%) and *C. glabrata* (19%). Average patient age was 65 years and majority were of Hispanic descent (49%) with common comorbidities including diabetes (55% vs. 43%; p = 0.044) and chronic kidney disease (14% vs. 25%; p = 0.093) in *C. auris* vs. non-*C auris* groups, respectively. More *C. auris* patients required mechanical ventilation (86% vs. 63%; p = 0.019), and had central lines placed (93% vs. 67%; p = 0.002). All *C. auris* isolates were susceptible to echinocandins but resistant to fluconazole. *C. auris* patients had a non-significantly higher crude rate of in-hospital mortality (62% vs. 49%; p = 0.075) with a longer mycological eradication time (mean 5 vs. 3 days; p = 0.196). Median time to mortality was longer in *C. auris* patients (76 days vs. 30 days, p < 0.0006). These patients also had longer hospital stays (median 111 vs. 40 days, p < 0.002) and nonsignificant higher recurrence rates (14% vs. 6%; p = 0.091). More *C. auris* survivors were discharged to long-term care facilities (78% vs. 56%).

**Conclusion:**

Patients with C. auris fungemia had a significantly longer median time to mortality and LoS, in addition to a non-significantly higher rate of recurrence and mortality.

**Disclosures:**

**All Authors**: No reported disclosures

